# Silk Fibroin Hydrogels Incorporated with the Antioxidant Extract of *Stryphnodendron adstringens* Bark

**DOI:** 10.3390/polym14224806

**Published:** 2022-11-08

**Authors:** Vivian P. de Brito, Maurício M. de Souza Ribeiro, Juliane Viganó, Mariana A. de Moraes, Priscilla C. Veggi

**Affiliations:** 1 Department of Chemical Engineering, Institute of Environmental, Chemical and Pharmaceutical Sciences, Federal University of São Paulo, Diadema 09913-030, SP, Brazil; 2 Nature Science Center, Federal University of São Carlos, Rod. Lauri Simões de Barros, Km. 12-SP 189, Buri 18290-000, SP, Brazil

**Keywords:** antioxidant activity, biopolymer, high-performance dressings, medicinal plant, phenolic compounds, tannins

## Abstract

*Barbatimão* (*Stryphnodendron adstringens*) is a Brazilian medicinal plant known for its pharmacological properties, including healing activity related to its phenolic composition, which is chiefly given by tannins. In order to preserve its stability and bioactivity, *barbatimão* extracts can be incorporated into (bio-)polymeric matrixes, of which silk fibroin stands out due to its versatility and tunable properties. This work aimed to obtain *barbatimão* bark extract rich in phenolic compounds and evaluate its incorporation in fibroin hydrogels. From the extraction process, it was observed that the PG (propylene glycol) extract presented a higher global yield (X_0_) and phenolic compounds (TPC) than the ET (ethanol) extract. Furthermore, the antioxidant activity (ORAC and FRAP) was similar between both extracts. Regarding the hydrogels, morphological, chemical, thermal, and mechanical characterizations were performed to understand the influence of the *barbatimão* extract and the solvent on the fibroin hydrogel properties. As a result, the hydrogels containing the *barbatimão* PG extract (BT/PG hydrogels) showed the better physical–chemical and structural performance. Therefore, these hydrogels should be further investigated regarding their potential in medical and pharmaceutical applications, especially in wound healing.

## 1. Introduction

Using plants as a therapeutical resource is a technique that has been applied for centuries. Brazil, which has the largest biodiversity in the world, has attracted the attention of researchers and pharmaceutical industries in the studies of native plants and their mechanism of action [1]. One of these plants widely studied is *Stryphnodendron adstringens*, well known as *barbatimão*. This native plant from the Brazilian Cerrado biome has a well-spread therapeutical use. The bark of this species is used as a wound-healing and anti-inflammatory agent [2]. Additionally, studies have proved its antimicrobial, antiprotozoal, antifungal, antiviral, and anticancer properties [3,4,5,6].

The pharmacological properties of *barbatimão* are associated with its high concentration of phenolic compounds, mainly tannins varying between 30 and 35% by mass. Tannins are a sub-class of phenolic compounds obtained mainly from plant matrices soluble in water. However, they can sequester free radicals, complex metal ions, and other macromolecules [7], becoming bioactive with great potential for the pharmaceutical, cosmetic, and food industries. Conventional extraction methods have obtained *barbatimão* extracts, such as static and dynamic maceration and percolation [2,8,9]. However, ultrasound emerged as an alternative economic, efficient, and simple extraction method to obtain bioactive compounds [10].

Likewise, tannins can be incorporated into synthetic or natural polymeric matrices to preserve their properties. Natural polymers have become increasingly attractive due to their environmental appeal, faster degradation than synthetic polymers, and good potential for use as a biomaterial [11]. Silk fibroin produced by the silkworm *Bombyx mori* is a biopolymer well-studied for biomaterial applications since it has good blood compatibility and permeability to oxygen and water vapor [12]. Because of these factors, this biopolymer may be suitable for tissue regeneration, bone reconstruction scaffolds, and wound dressings [13]. In addition, silk fibroin can be molded in different forms, such as films, microcapsules, and hydrogels [13,14]. Hydrogels have been used in the medical field since the 1960s because of their advantages as drug carriers, mimicking the extracellular matrix and biocompatibility [15]. In addition, there are studies on incorporating bioactive substances, such as curcumin and Aloe vera, in silk fibroin hydrogels and films to assess the healing time in wounds and the controlled release in scaffolds [16,17,18]. However, there are few studies on incorporating *barbatimão* extract in biopolymer matrices. Oliveira Mori et al. [19] incorporated extracts of *barbatimão* bark into zein nanofibers obtained by electrospinning and studied the properties and morphology. Nascimento et al. [20] evaluated the properties and morphology of alginate-based films incorporated with extracts of *barbatimão* bark in free and microencapsulated form. Thus, there are no studies in the literature on the incorporation of *barbatimão* bark extract in fibroin-based hydrogels. In this context, the present work proposed to obtain extracts from the *barbatimão* bark and study their incorporation in silk fibroin hydrogels. Extracts were evaluated in terms of phenols and tannins content and antioxidant activity. In addition, the hydrogels were characterized regarding morphological, chemical, mechanical, and thermal properties.

## 2. Materials and Methods

### 2.1. Chemicals

Ethanol (Dinâmica, Indaiatuba, SP, Brazil), distilled water, and propylene glycol (Synth, Diadema, SP, Brazil) were used as solvents. DNS (dinitrosalicylic acid, Synth, Diadema, SP, Brazil), sodium hydroxide (Synth, Diadema, SP, Brazil), potassium tartrate (Dinâmica, Indaiatuba, SP, Brazil), sodium tetrahydrate (Dinâmica, Indaiatuba, SP, Brazil), and glucose (Dinâmica, Indaiatuba, SP, Brazil) were used for total reducing sugar content analysis. Tannic acid (Synth, Diadema, SP, Brazil) was used for total phenolic content analysis. Folin-Denis reagent (Dinâmica, Indaiatuba, SP, Brazil) and sodium carbonate (Dinâmica, Indaiatuba, SP, Brazil) were used for total phenolic and tannin content and the preparation of silk fibroin aqueous solution. Casein and pyrogallol (Dinâmica, Indaiatuba, SP, Brazil) were used for total tannin content analysis. Sodium buffer solution (pH 3.6, Dinâmica, Indaiatuba, SP, Brazil), TPTZ (2,4,6-tris (2-pyridyl)-s-triazine, Sigma-Aldrich, SP, Brazil), HCl (Synth, Diadema, SP, Brazil), FeCl_3_ (Dinâmica, Indaiatuba, SP, Brazil), Trolox (6-hydroxy-2,5,7,8-etramethylchromane-2-carboxylic acid, Sigma-Aldrich, Barueri, SP, Brazil), potassium phosphate buffer solution (pH 7.4, Quimilab, Jaboatão dos Guararapes, PE, Brazil), fluorescein (Sigma-Aldrich, Barueri, SP, Brazil), and AAPH (2,2′-azobis (2-methylpropionamidine) dihydrochloride), (Sigma-Aldrich, Barueri, SP, Brazil) were used for antioxidant capacity analysis. Calcium chloride (Synth, Diadema, SP, Brazil) was used to prepare silk fibroin aqueous solution.

### 2.2. Barbatimão Bark Preparation

The *barbatimão* barks were donated by Kampo de Ervas (Ribeirão Preto, SP, Brazil) in March 2019 (batch number: 08032019). The barks were shade-dried at room temperature and sent to the laboratory in black plastic bags. The dried *barbatimão* barks were shredded in a shredder (Lippel, Agrolândia, SC, Brazil) to reduce the size and then ground in a knife mill (Marconi, Piracicaba, SP, Brazil). The samples were fractionated in a vibrating system (Telastem, São Paulo, SP, Brazil) using 0.710 and 0.106 mm sieves. The resulting material was stored at −18 °C and protected from light and moisture. The raw material was characterized by moisture content, drying in an oven at 105 °C until constant mass, total ash, with a muffle furnace at 450 °C until constant mass, and pH. The moisture and total ash content, and pH of *barbatimão* barks samples were 12.00 ± 0.04%, 1.76 ± 0.07%, and 4.75 ± 0.09, respectively.

### 2.3. Obtaining Barbatimão Extracts

The *barbatimão* extracts were obtained through two extraction methodologies, ultrasonic bath (Eco-sonics, Indaiatuba, SP, Brazil, 37 kHz frequency) and agitated bath (Logen, Diadema, SP, Brazil). The ultrasound method was applied since it is well-known as an environmental technology that uses short process time and ecological solvents [21]. The extraction procedure was adapted from Sousa et al. [22], considering the optimized parameter regarding phenolic compounds for *barbatimão* barks. Approximately 15 g of *barbatimão* powder was weighed for the extractions, and 150 mL of 65% (*v*/*v*) hydroalcoholic solution was added. The samples remained in the ultrasonic bath for 30 min (ET extract).

Mechanical agitation bath extraction was performed as a conventional technique that uses propylene glycol as a solvent, widely used by the pharmaceutical industry. The extractions were performed according to Baurin et al. [23] regarding the strong antioxidation power of *barbatimão* barks. Approximately 15 g of *barbatimão* powder was mixed with 150 mL of 50% (*v*/*v*) water and propylene glycol solution, which was agitated for 120 min at 45 °C (PG extract). All extractions were performed in triplicate, and the extracts were vacuum-filtered with a qualitative cellulose filter and stored at −18 °C in absence of light.

### 2.4. Extract Characterization

#### 2.4.1. Total Phenolic Content (TPC)

TPC was determined according to the Folin-Denis method described by Campos et al. [24] with some modifications. The calibration curve (0.05–0.4 mg/mL) was obtained with tannic acid. For TPC determination, 7 mL of distilled water and 0.5 mL of Folin-Denis reagent were added in test tubes containing 0.4 mL of each liquid extract diluted solution. The mixture was agitated in a tube shaker (Gehaka, model Magic, São Paulo, SP, Brazil) and let stand for 3 min. After this time, 1 mL of the saturated sodium carbonate solution was added, and the final volume was adjusted to 10 mL by adding distillate water. The absorbance was read in a spectrophotometer (Thermo Scientific, model Genesys 10S, Waltham, Massachusetts, EUA) at 730 nm exactly 30 min after the basis was added. Each sample was analyzed in duplicate. The results were expressed in terms of mg of equivalent tannic acid (ETA) per mL of extract.

#### 2.4.2. Total Tannins Content (TTC)

TTC in the extracts was determined as described by Farmacopéia Brasileira [25] with some modifications. The calibration curve was built with pyrogallol as standard at different concentrations (0.01–0.045 mg/mL). First, the total polyphenols were determined. An aliquot of 0.25 mL of diluted extract was added to 0.25 mL of the Folin–Denis reagent and 2 mL of the sodium carbonate solution (the same prepared for TPC, in Section 2.4.1). The absorbance was measured at 730 nm in a spectrophotometer (Thermo Scientific, model Genesys 10S, Waltham, Massachusetts, EUA) 2 min after adding the sodium carbonate. Next, a suspension consisting of 20 mL of the diluted extract with 0.3 g of casein was stirred for 60 min to determine the non-tannin fraction. After casein precipitation, the suspension was filtered (quantitative filter paper with a pore size of 25–40 µm). An aliquot of 0.25 mL of the filtrate solution was added to 0.25 mL of the Folin–Denis reagent and 2 mL of the sodium carbonate solution. The reading in the spectrophotometer was performed as described to determine the total polyphenols step. The total tannin content was calculated as the difference between the total polyphenols and non-tannin fractions. Each sample was analyzed in duplicate. The results were expressed in mg of equivalent pyrogallol (EP) per mL of extract.

#### 2.4.3. Ferric Reducing Ability Power (FRAP)

The ability to reduce iron was assessed based on the FRAP method [26]. The FRAP solution was obtained by adding 0.3 mol/L sodium buffer solution (pH 3.6), 10 mmol/L TPTZ solution in 40 mmol/L HCl solution, and 20 mmol/L FeCl_3_ solution in the proportions of 10:1:1, respectively. This solution was prepared immediately before analysis and warmed at 37 °C. Then, 25 μL of liquid extract diluted in distilled water was added to 175 μL of FRAP solution and protected from light for 30 min at 37 °C. This procedure was performed in triplicate, diluting the samples 1000 times. The absorbance was read at 595 nm in a microplate reader (BMG LABTECH GmbH, Ortenberg, Germany) with Omega Mars 3.32R5 data analysis software. Trolox (0.01–0.06 mg/mL) was used to obtain the standard curve. The results were expressed in mg of Trolox equivalent (TE) per mL of extract.

#### 2.4.4. Oxygen Radical Absorbance Capacity (ORAC)

The hydrophilic ORAC method (h-ORAC) was determined according to Ou et al. [27] with adaptations. First, the samples (5–25 µg/mL) were diluted in 75 mmol/L potassium phosphate buffer solution (pH 7.4). A potassium phosphate buffer solution was used as a blank. Next, 25 µL of the sample, which was diluted 1000 times, standard or white, and 150 µL of fluorescein was added to each silver microplate well. Then, the micro-plates were incubated in a FLUOstar Omega microplate reader BMG LABTECH GmbH (Ortenberg, Germany) at 37 °C for 15 min, followed by the addition of 25 µL of AAPH solution to each well. The fluorescence reduction (excitation at 485 nm and emission at 510 nm) was measured for 100 min at 37 °C in a microplate reader. This procedure was performed in triplicate. The results were expressed in mg of TE per mL of extract.

#### 2.4.5. Total Reducing Sugar Content (TRSC)

Total reducing sugars were determined using the dinitrosalicylic acid method (DNS) [26]. First, a solution was prepared by mixing 0.25 g of DNS in 5 mL of 2 M sodium hydroxide solution. Then, a second solution was prepared under heating by mixing 7.5 g of potassium sodium tartrate tetrahydrate in 12.5 mL of distilled water. These two solutions were mixed, and their volumes were augmented to 25 mL with distilled water. Finally, the extracts were diluted in distilled water 100 times, and the standard curve was obtained using dilutions of glucose (0.2–1.6 mg/mL).

The reactions were carried out in test tubes by mixing the extract, the blank, or the calibration curve in the DNS reagent in the proportion of 1:1 (*v*:*v*). The test tubes were shaken for 10 s and heated for 15 min in boiling water. The tubes were then cooled in an ice bath, and distilled water was included in each tube in a 1:1:8 (*v*:*v*:*v*) ratio. Next, the tubes were shaken, and 200 µL of each sample was transferred to 96-well microplates. The absorbance was read at 540 nm in a microplate reader (BMG LABTECH GmbH, Ortenberg, Germany). This procedure was performed in triplicate, and the results were expressed in mg of glucose per mL of extract.

### 2.5. Preparation of Silk Fibroin Aqueous Solution

Silkworm cocoons from *Bombyx mori* were supplied by the silk company Bratac (Londrina, PR, Brazil). The preparation of silk fibroin aqueous solution from silkworm cocoons followed the methodology proposed by Nogueira et al. [28]. First, for sericin removal, silkworm cocoons were degummed three times by soaking in 1 g/L sodium carbonate (Na_2_CO_3_) solution at 85 °C for 30 min. Next, the fibroin fibers were washed in abundant distilled water and left to dry at room temperature for 48 h. Then, 10 g of the dried fibers were dissolved in 200 mL of a ternary solvent of calcium chloride, ethanol, and water (CaCl_2_:CH_3_CH_2_OH:H_2_O 1:2:8 molar) at 85 °C for 90 min. The 5% (*w/v*) fibroin saline solution was then dialyzed in distilled water using a cellulose membrane of 3.5 kDa molecular weight cut-off (SnakeSkin Dialysis Tubing, Thermo Scientific, Waltham, Massachusetts, EUA) for three days at 10 °C to remove the salts of the solvent and obtain an aqueous solution of fibroin. The dialysis water was changed every 24 h, keeping the ratio of fibroin solution to water at 1:15. Finally, a 1.6% (*w*/*v*) aqueous solution of fibroin was obtained, which was determined gravimetrically by drying the fibroin aqueous solution in a Petri dish.

### 2.6. Incorporation of the Extracts into the Silk Fibroin Hydrogel

*Barbatimão* extracts were diluted ten times in ethanol and incorporated in a 1:11 volumetric ratio into a fibroin solution. Preliminary tests defined this proportion. In addition, hydrogels with only the solutions used in the extractions (ethanol or propylene glycol) were prepared for comparative control. Table 1 presents the nomenclature and description of the hydrogels studied. The extracts were slowly added to the fibroin solution and gently mixed for 15 min in a magnetic stirrer to prepare the hydrogels. Then, the mixture was placed in a cylindrical mold and kept in a thermostatic bath at 37 °C until gelation.

### 2.7. Hydrogel Characterization

#### 2.7.1. Scanning Electron Microscopy (SEM)

This technique was used to verify the morphology of the hydrogels by scanning the sample surface with an electron beam. The samples were frozen with liquid nitrogen, fractured, and lyophilized for 24 h and covered with gold for SEM. A Leo 440i (LEO Electron Microscopy, Cambridge, UK) was used with 10 kV voltage and 50 mA current. The pore sizes were measured using the software ImageJ^®^ through the measurement of 20 pores in each image.

#### 2.7.2. Fourier Transform Infrared Spectroscopy (FTIR) 

FTIR analysis was performed on the hydrogels in an Agilent Cary 630 (Santa Clara, Califórnia, EUA) in ATR mode to verify the secondary structure of silk fibroin. The spectra were obtained in the range from 650 to 4000 cm^−1^, with 4 cm^−1^ and 128 scans resolution.

#### 2.7.3. Thermogravimetry Analysis (TGA)

TGA was performed in a thermogravimeter model TGA/DSC1 from Mettler (Schwerzenbach, Switzerland) to assess the behavior and thermal stability of the hydrogels. To accomplish this, the samples were frozen with liquid N_2_ and lyophilized for 24 h. Then, they were analyzed in a temperature range from 25 °C to 600 °C, with a rate of 10 °C/min and an N_2_ flow rate of 50 mL/min.

#### 2.7.4. Compressive Strength

Compressive strength was performed on cylindrical hydrogels with approximately 25.4 mm diameter and 21.7 mm height. The test was performed on a texture analyzer CT3 (Brookfield Engineering, Middleboro, MA, USA) with a cylindrical geometry of 40 mm in diameter, a 50 kg load cell, and at room temperature. The mechanical properties were obtained from the compression of the hydrogels to 80% of the original height. A compression rate of 1 mm/s was used. Tests were performed on four samples of each hydrogel. The Hencky stress and strain were calculated according to Equations (1) and (2), where F(t) is the force (N) in a specific time t (s), A_0_ is the initial cross-section of the sample (m^2^), L_0_ is the initial length (m), and L(t) is the length (m) in a specific time t (s).

(1) $$\mathrm{Hencky}\;\mathrm{stress}\;={\;F}_{\left(t\right)}\cdot L_{\left(t\right)}/\left(L_{0}\cdot A_{0}\right)$$

(2) $$\mathrm{Hencky}\;\mathrm{strain}\;=-\;\ln\left(L_{\left(t\right)}/L_{0}\right)$$

#### 2.7.5. Rheological Tests

The rheological tests were carried out in a rheometer (MCR92, Anton Paar, Graz, Austria) with a 20 mm diameter flat plate geometry, with a gap of 1 mm and a temperature of 25 °C to determine the elastic and viscous modules of the hydrogels. The samples were prepared in a cylindrical mold and were approximately 25.4 mm in diameter and 21.7 mm in height. The analysis was performed in duplicate, and the samples were cut to approximately 10 mm in thickness. First, a stress scan was performed on the samples to identify the region of linear viscoelasticity. Then, this determined voltage was used in the frequency variation test, which was conducted in a range from 1 to 100 Hz.

### 2.8. Statistical Analyses 

The results were compared by the Tukey test using the software Minitab^®^ (State College, PA, EUA), and the differences were considered statistically significant at a significance level of *p* < 0.05.

## 3. Results and Discussion

### 3.1. Extract Characterization

**Figure 1:**
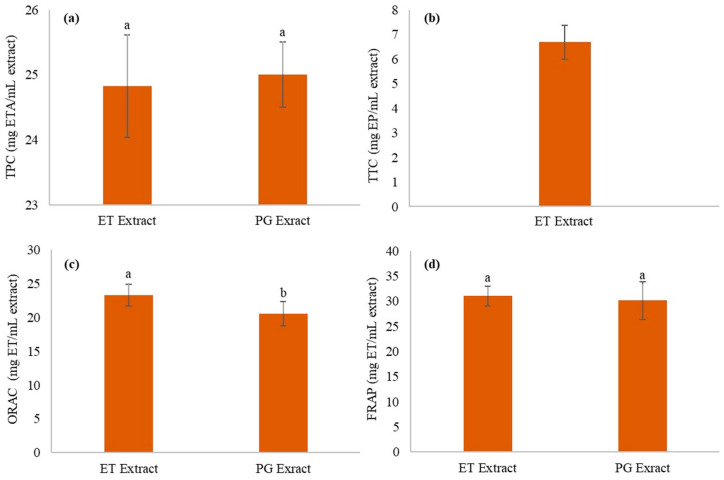
Comparison of (**a**) total phenolic concentration, (**b**) total tannin concentration, antioxidant activity by (**c**) ORAC, and (**d**) FRAP from extractive processes using ethanol and propylene glycol. Equal a,b letters in the same column indicate no statistically significant difference between the averages, assessed by the Tukey test at the 95% level of significance.

The results of TPC and TTC for ET and PG extracts are presented in Figure 1a,b. It can be observed that the TPC (25.00 ± 0.51 mg ETA/mL extract) of the PG extract was similar to the ET extract (24.83 ± 0.79 mg ETA/mL extract). These results indicate that both processes had similar performance for the extraction of phenolic compounds. Regarding the TTC of the PG extracts, the non-tannin fraction presented a higher absorbance than the total phenolic compounds fraction, resulting in an unreal TCC (negative concentration); for this reason, Figure 1b does not present TTC for PG extract. On the other hand, the ET extract presented a TTC of 6.69 ± 0.69 mg of EP/mL of extract. The result obtained for the PG extract may be related to reaction time, stirring frequency, and even the amount of casein used [7]. Moreover, this complexation process of tannins with proteins is, in general, a reversible process [7]. Therefore, long-time intervals between the complexation and the absorbance reading would collaborate with the decomplexation and, consequently, interfere with the final absorbance value of the non-tannin fraction.

The binding process of tannins to protein and their subsequent precipitation is a multi-stage process. Initially, the tannins bind to the protein by a reversible hydrophobic process, forming a soluble structure. Then, cross-linking with the peptides occurs with excess tannins, making the structure insoluble (involvement of the protein by tannins). In the end, further aggregation of these insoluble complexes occurs, increasing the particle size [29,30,31]. This complex is responsible for the samples’ turbidity and consequent increase in absorbance [32], which justifies the methodological need to separate these structures by filtration in non-tannin fraction. These complexations of proteins by tannins can also generate structures with particle sizes smaller than 100 nm, increasing the sample’s turbidity by increasing the mass density [32]. High particle size complexes with high precipitation are formed when there are proportional amounts between the proteins and tannins. Smaller structures will form if there is an imbalance between the available amounts of protein and tannin [30]. This phenomenon could justify the result observed by the PG extract. However, the turbidity of the sample was not observed after filtration. Still, some structures with smaller particle sizes could have passed through the filter, resulting in a higher absorbance result. Considering that the amount of casein was fixed between the samples, this result would indicate that the PG extract could have a lower quantity of tannins than the ET extract, which would have generated smaller particles that ended up passing through the filter. Another factor contributing to the increased absorbance of the non-tannin fraction is the presence of polysaccharides in the extract, which could interfere with this analysis in two ways; first, the formation of ternary complexes with the protein–tannin complex, generating a soluble compound. Second, the polysaccharides could be complex with the tannins, generating soluble structures and competing with the proteins. In both cases, separating the tannins in the non-tannin fraction would not be possible, resulting in higher absorbances [31].

Ardisson et al. [33] found, using a sequential extraction process by static maceration followed by percolation with mixtures of PG in water, that the highest yield in the extraction of tannins from *barbatimão* bark was the mixture of 80% PG in water. These authors also extracted tannins in all tested solvents (70, 80, and 90%), which indicates that propylene glycol is a solvent capable of extracting tannins. However, the concentration of this solvent in mixtures with water can affect the composition of the extract. Therefore, with the highest amount of water in the extraction solvent, other phenolic compounds were extracted in addition to tannins.

The difference in phenolic compound content with that found in the literature may be due to climatic factors, type of solvents, extraction method, or even the harvest region or season of the *barbatimão*. For example, Santos et al. [34] observed in their studies that the month of harvesting the *barbatimão* changes its chemical composition and, therefore, the phenolic compounds. In addition, biotic and abiotic factors can also interfere with the biosynthesis of phenolic compounds [35].

Figure 1 also shows the antioxidant capacity of the extracts by the ORAC (Figure 1c) and the FRAP (Figure 1d) methods. It is possible to observe that for the ORAC method, ET extract showed higher antioxidant activity than the PG extract (23.30 ± 1.61 and 20.52 ± 1.79 mg TE/mL extract, respectively), while for the FRAP method, there was no statistically significant difference (PG: 30.11 ± 3.73 mg TE/mL extract and 31.00 ± 1.94 mg TE/mL extract). These results indicate that the antioxidant activity of the extracts may be related to compounds other than phenolic compounds, which differs from the literature that correlates the phenolic compounds with the antioxidant activity [36]. Few studies evaluated the antioxidant capacity of *barbatimão* bark extract using ORAC and FRAP techniques. It is worth noting that the precise comparison with those in the literature is difficult since the extracts’ phenolic composition can be different due to the difference in the extraction technique and the issues inherent in the raw material itself and its harvesting process.

The total reducing sugar content observed in the extracts of *barbatimão* was similar (ET: 21.92 ± 2.59 mg glucose/mL extract and PG: 19.38 ± 2.40 mg glucose/mL extract) with no statistically significant difference between the values of both extracts. Reducing sugars in the extract is not desired since they reduce bioactive molecules’ concentration. Furthermore, compared with grapes that are rich in tannins (185.26 ± 1.74 to 202.67 ± 1.30 mg glucose/mL extract) [37], the ET and PG extracts obtained in the presented work are substantially lower.

### 3.2. Hydrogel Characterization

The ET and PG extract, the hydroalcoholic solution of 65% (*v*/*v*), and the propylene glycol solution of 50% (*v*/*v*) in water were incorporated into the silk fibroin hydrogels. The approximate gelation times were obtained by the tube inversion method, which is the moment that the solution is no longer in the liquid state. As a result, PG hydrogel showed a longer gelation time (approximately one and a half days). In contrast, BT/ET hydrogel and ET hydrogel were already gelled after 12 h in the bath, and BT/PG hydrogel after 18 h.

The silk fibroin gelation can be affected by several factors, such as pH, temperature, or salt concentration [38,39,40]. Jing et al. [41] studied the incorporation of tannic acid in silk fibroin hydrogels. They observed that the gelation time of hydrogels with tannic acid was 11 h, much shorter than that of the hydrogel without this substance, which was still in the liquid phase after 30 days. This accelerated sol–gel transition occurs due to the tannins’ ability to complex with macromolecules and, therefore, favor molecular interactions and induce gelation.

**Figure 2:**
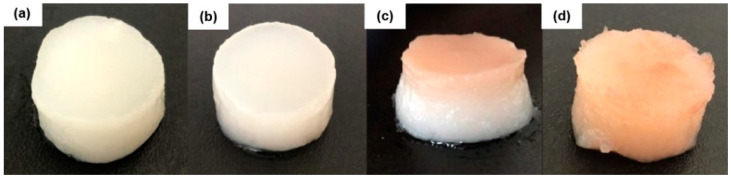
Photographs of the hydrogels ET (**a**), PG (**b**), BT/ET (**c**), and BT/PG (**d**).

Figure 2 shows the physical aspect of the hydrogels. In general, the hydrogels kept the cylindrical shape of the mold. The hydrogels containing *barbatimão* extracts (BT/ET and BT/PG) showed a brown color, demonstrating the incorporation of the extract, while the hydrogels ET and PG were uniform and white, which is a typical aspect of fibroin hydrogels. The PG hydrogel (Figure 2b) lost structural stability at room temperature after some days of gelation, which restrained the SEM analysis of this hydrogel (see Section 3.3). In addition, the hydrogel BT/ET (Figure 2c) has shown weak structural stability, releasing more water from its structure than the other hydrogels and showing a non-uniform distribution of the extract, which was more concentrated at the top of the hydrogel.

### 3.3. Scanning Electron Microscopy (SEM)

**Figure 3:**
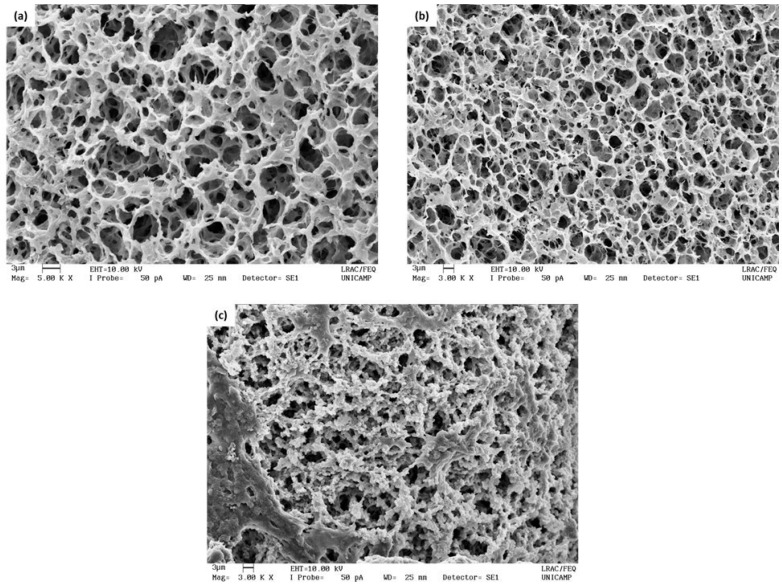
Scanning electron microscopy (SEM) images of hydrogels at 5000× magnification: (**a**) ET hydrogel, (**b**) BT/ET hydrogel, and (**c**) BT/PG hydrogel.

The SEM images of the hydrogels are shown in Figure 3. The PG hydrogel sample was not analyzed since it returned to the solution state. This reversible process of the sol–gel transition may have occurred because this hydrogel did not form a stable β-sheet structure, as confirmed in the FTIR analysis (shown in Section 3.4). Therefore, it likely returned to the solution state due to an increase in temperature during SEM sample transportation and preparation.

It is possible to observe that the ET (Figure 3a) and BT/ET hydrogels (Figure 3b) had similar porous structures, with pore diameters of 3.270 ± 0.602 μm and 3.393 ± 0.473 μm, respectively. On the other hand, the BT/PG hydrogel (Figure 3c) showed thicker walls and, therefore, a smaller pore size of 2.340 ± 0.614 μm. The thickening of the walls likely occurred due to the interaction of tannins with fibroin, contributing to a more compact structure and changing its mechanical properties. These results agree with those reported by Jing et al. [41], which found that increasing the tannic acid concentration from 0.1 to 0.7% (*w*/*w*) in silk fibroin hydrogels results in the formation of more compact and cross-linked structures and, consequently, smaller pore sizes (from 43 to 26 μm).

### 3.4. Fourier Transform Infrared Spectroscopy (FTIR)

**Figure 4:**
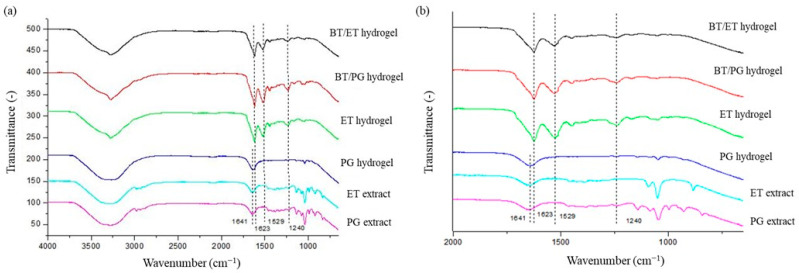
FTIR spectra of hydrogels and *barbatimão* extracts in the 4000 a 650 cm^−1^ region (**a**) and amplification in the region from 2000 to 650 (**b**).

Figure 4 shows the FTIR spectra of the hydrogels and *barbatimão* extracts. FTIR is a valuable technique to detect fibroin conformation (silk I or silk II). All spectra showed a band referring to the -OH stretching between 3274 and 3290 cm^−1^, which indicates the presence of water. For the BT/ET, BT/PG, and ET hydrogels, there are amide I and II bands in the wavelengths of 1623 cm^−1^ and 1529 cm^−1^, respectively. These bands characterize the silk II conformation [42]. In addition, amide III is present in 1240 cm^−1^, which is an intermediate value between silk I (1230 cm^−1^) and silk II (1260 cm^−1^), indicating that there are conformations of silk I and II, with the predominance of the latter [43].

The incorporation of ET extract in the hydrogel (BT/ET hydrogel) did not affect the conformation of the fibroin nor the gelation time compared with the ET hydrogel since both show the same peak profile in the FTIR.

For the PG hydrogel, there is a band referring to amide I at 1641 cm^−1^, which indicates the presence of the conformation structure of a random coil [44]. This hydrogel has no other peaks related to amides II and III, indicating major random conformation. However, when evaluating the BT/PG hydrogel, it is possible to observe a reduction in the gelation time and a change in the conformation of fibroin when compared with the PG hydrogel. These results indicate a transition from the silk I structure to a silk II structure, possibly because of the capacity of tannins to complex with other proteins through hydrogen bonds and hydrophobic interactions [45].

These results related to the presence of tannins in fibroin hydrogels can be explained since organic solvents, such as ethanol, favor fibroin’s silk I quick conformational transition into silk II [39]. Therefore, the presence of tannins does not influence the conformational structure of BT/ET hydrogel compared with ET hydrogel. On the other hand, the introduction of propylene glycol in the fibroin hydrogel (PG hydrogel) does not influence the conformational transition of fibroin, which retains the unstable silk I structure. Therefore, the transition from silk I to silk II in the BT/PG hydrogel is likely related to the presence of tannins in the PG extract.

Jing et al. [41] observed a similar effect when analyzing fibroin hydrogels containing tannic acid. They concluded that the presence of tannic acid has favored the formation of stable β-sheets (silk II), improved mechanical properties, and showed antimicrobial and antioxidant properties.

### 3.5. Thermogravimetry (TGA)

**Figure 5:**
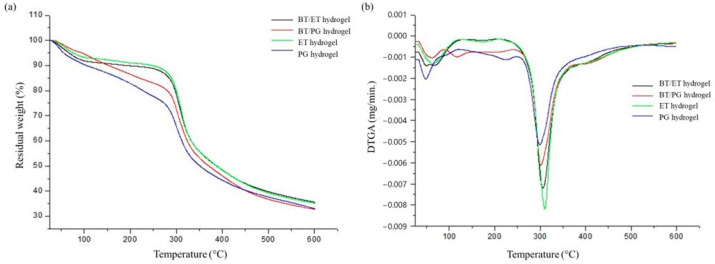
(**a**) Thermogram and (**b**) first derivative thermograms (DTGA) of the hydrogels.

The thermogram of the hydrogels is shown in Figure 5, and it is possible to observe that the ET and BT/ET hydrogels have a similar thermal profile, with only a small decrease in thermal stability in the hydrogel with *barbatimão* extract. Furthermore, these hydrogels containing ethanol (ET and BT/ET) also had a mass loss lower than those of the propylene glycol hydrogels (PG and BT/PG) in almost all temperature ranges, demonstrating higher thermal stability. This is possibly caused by ethanol, which is a fibroin crosslinker.

BT/PG hydrogel had higher thermal stability than PG hydrogel, likely due to the strong interaction between propylene glycol, tannins, and silk fibroin. 

The first thermal event in all hydrogels occurred between 33.16 °C and 99.02 °C and was related to water evaporation [42]. For hydrogel PG and BT/PG, there was a higher loss of mass, which extended to approximately 240 °C and, possibly, was due to propylene glycol evaporation [46]. The main thermal event for the hydrogels occurred between 250 °C and 350 °C and was related to the thermal degradation of silk fibroin [42]. The thermal decomposition between 400 °C and 600 °C was due to the carbonization of silk fibroin [47]. These results are similar to those found by Ramírez [48], who studied the influence of aging of silkworm cocoons in fibroin films and found three important thermal events related to water evaporation, degradation, and carbonization of silk fibroin.

### 3.6. Compressive Strength

**Figure 6:**
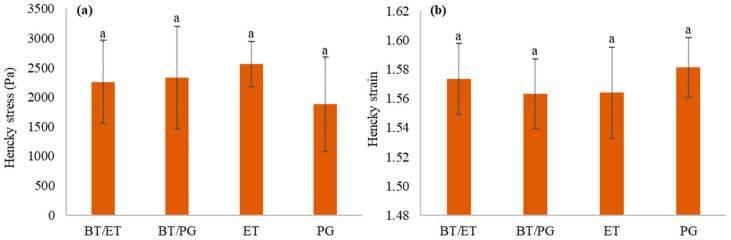
(**a**) Hencky stress and (**b**) Hencky strain of different hydrogels. Equal a letters in the same column indicate no statistically significant difference between the averages, assessed by the Tukey test at the 95% level of significance.

The Hencky stress and strain values are shown in Figure 6. There was no statistically significant difference between the hydrogels. Therefore, the incorporation of *barbatimão* extracts did not affect the mechanical properties of fibroin hydrogels.

Although the BT/PG hydrogel had a smaller pore size than BT/ET and ET hydrogels, and was, therefore, expected to have greater compressive strength, the Hencky stress values between these hydrogels showed no statistical difference. This behavior is different from that in Kim et al. [38], which found that the decrease in the pore size of silk fibroin scaffolds resulted in a better distribution of the applied strength, reducing the propagation force and increasing the compressive strength of the biopolymer matrices.

The values of Hencky stress are consistent with the literature for fibroin hydrogels containing high amounts of organic solvents, such as ethanol [49]. Several factors may influence the mechanical properties of silk fibroin hydrogels, such as fibroin concentration [50] and the addition of organic solvents, typically used to accelerate fibroin sol–gel transition [49]. Typically, hydrogels present low mechanical strength due to their high porosity and high water content.

### 3.7. Rheological Tests

**Figure 7:**
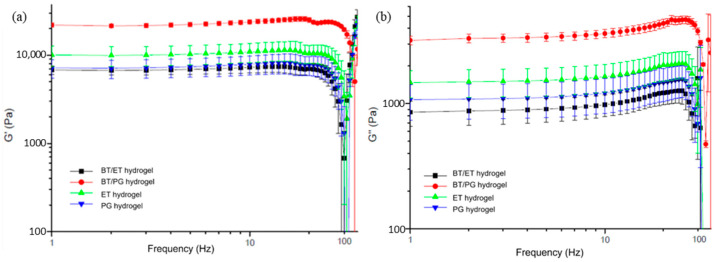
Rheological curve of (**a**) storage (G′) and (**b**) loss (G″) modulus of the hydrogels.

The storage (G′) and loss (G″) moduli obtained in the rheological test of the hydrogels are shown in Figure 7. The incorporation of *barbatimão* extracts did not change the rheological behavior of the hydrogels since the same profile was observed; however, there were changes in the values of G′ and G″. The G′ values were higher than G″ for all samples, and during the entire analysis, the hydrogels presented a low dependency of the frequency. These factors prove the viscoelasticity of the samples, and the rheological profile is characteristic of gels with high internal structural strength [51].

It is possible to observe that the incorporation of the PG Extract (BT/PG hydrogel) increased both moduli when compared with hydrogel values with only propylene glycol (PG hydrogel). Therefore, there was an improvement in the resistance and elasticity of the hydrogel with *barbatimão* extract, which formed a more cross-linked structure than the hydrogel with only propylene glycol, leading to an increase in the moduli. Furthermore, Ma et al. [52] studied silk fibroin films containing tannins. They concluded that incorporating these bioactive substances in the structure of the films improves their mechanical properties since the tannins can complex with other proteins through hydrogen bonds and hydrophobic bonds, creating a more stable structure than those present in films without tannins.

However, the opposite effect was observed in the incorporation of ET extract. The G′ and G″ modulus values decreased compared with the hydrogel with ethanol. The structure formed between the hydrogel and the extract is less stable than the one formed in the hydrogel with only ethanol. Pham et al. [53] observed a similar behavior in chitosan hydrogels, showing lower storage moduli in the hydrogels containing curcumin.

## 4. Conclusions

This work studied two different methodologies to obtain *barbatimão* extracts and incorporate these extracts into silk-fibroin-based hydrogels to understand the influence of the solvent and the extracts on the properties of fibroin hydrogels. It was observed that both extracts (ET and PG) presented similar behavior in TPC, antioxidant capacity, and total reducing sugar content. In addition, the ET, BT/ET, and BT/PG hydrogels presented a predominance of the silk II conformation. In contrast, the PG hydrogel exhibited silk I conformation and weak structural stability, undergoing a gel–sol transition after some days at room temperature. However, the incorporation of *barbatimão* extracts in the BT/PG hydrogels showed different properties when compared with the PG hydrogel, possibly due to the ability of tannins to complex with proteins. As a result, BT/PG hydrogel had a more compact structure, thermal stability, and better results in the rheological test.

The results achieved reinforce the innovation and contribution of this paper on understanding the interaction of *barbatimão* extracts and silk fibroin hydrogels for its future application in medical and pharmaceutical areas. As proposals for future work, the group points to the need for pharmacological study, in vivo and in vitro, of these hydrogels to verify their possible application as dressings involving wound-healing studies.

## Figures and Tables

**Table 1:** Nomenclature for hydrogels used in this manuscript.

Nomenclature	Description
ET hydrogel	Silk fibroin hydrogel containing ethanol solution at 65% (*v*/*v*)
PG hydrogel	Silk fibroin hydrogel containing propylene glycol solution at 50% (*v*/*v*)
BT/ET hydrogel	Silk fibroin hydrogel containing the *barbatimão* ethanolic extract
BT/PG hydrogel	Silk fibroin hydrogel containing the *barbatimão* propylene glycol extract
